# RP-HPLC/MS/MS Analysis of the Phenolic Compounds, Antioxidant and Antimicrobial Activities of *Salvia* L. Species

**DOI:** 10.3390/antiox5040038

**Published:** 2016-10-21

**Authors:** Hatice Tohma, Ekrem Köksal, Ömer Kılıç, Yusuf Alan, Mustafa Abdullah Yılmaz, İlhami Gülçin, Ercan Bursal, Saleh H. Alwasel

**Affiliations:** 1 Department of Chemistry, Faculty of Science, Erzincan University, Erzincan 24100, Turkey; htohma@erzincan.edu.tr (H.T.); koksalekrem@gmail.com (E.K.); 2 Department of Biology, Faculty of Science, Bingol University, Bingol 12000, Turkey; omerkilic77@gmail.com; 3 Department of Biology, Faculty of Science, Muş Alparslan University, Muş 49250, Turkey; y.alan@alparslan.edu.tr; 4 Department of Chemistry, Faculty of Sciences, Dicle University, Diyarbakır 21280, Turkey; mustafaabdullahyilmaz@gmail.com; 5 Department of Chemistry, Faculty of Science, Atatürk University, Erzurum 25240, Turkey; 6 Department of Zoology, College of Science, King Saud University, Riyadh 11451, Saudi Arabia; salwasel@ksu.edu.sa; 7 Department of Nursing, School of Health, Muş Alparslan University, Mus 49250, Turkey; ercanbursal@gmail.com

**Keywords:** *Salvia* L. species, antioxidant activity, antimicrobial activity, HPLC-MS/MS, phenolic compounds

## Abstract

The identification and quantification of the phenolic contents of methanolic extracts of three *Salvia* L. species namely *S. brachyantha* (Bordz.) Pobed, *S. aethiopis* L., and *S. microstegia* Boiss. and Bal. were evaluated using reverse phase high performance liquid chromatography, UV adsorption, and mass spectrometry (RP-HPLC/MS). In order to determine the antioxidant capacity of these species, cupric ions (Cu^2+^) reducing assay (CUPRAC) and ferric ions (Fe^3+^) reducing assay (FRAP) were performed to screen the reducing capacity and 1,1-diphenyl-2-picrylhydrazyl (DPPH) assay was employed for evaluation of the radical scavenging activity for both solvents. In further investigation, the antimicrobial activities of *Salvia* species were tested using the disc diffusion method against three Gram-positive and four Gram-negative microbial species, as well as three fungi species. The results showed that there is a total of 18 detectable phenols, the most abundant of which was kaempferol in *S. microstegia* and rosmarinic acids in *S. brachyantha* and *S aethiopis*. The other major phenols were found to be apigenin, luteolin, *p*-coumaric acid, and chlorogenic acid. All species tested showed moderate and lower antioxidant activity than standard antioxidants such as butylated hydroxyanisole (BHA), butylated hydroxytoluene (BHT), and ascorbic acid. The ethanolic extracts of *Salvia* species revealed a wide range of antimicrobial activity. *S. brachyantha* and *S. microstegia* showed the highest antimicrobial activities against *B. subtilis*, whereas *S. aethiopis* was more effective on *Y. lipolytica.* None of the extracts showed anti-fungal activity against *S. cerevisiae.* Thus these species could be valuable due to their bioactive compounds.

## 1. Introduction

Plants contain many bioactive phenolic compounds, which have biological activity including antioxidant and antimicrobial properties. They have many health benefits and can help in preventing some diseases [1,2]. Phenolic compounds are the main group of phytochemicals found in plants. They are widely distributed as second metabolites derived from phenylalanine or tyrosine amino acids [3,4]. The basic structure of phenols includes an aromatic ring and a hydroxyl group. Depending on the number of phenolic units, the location and the number of hydroxyl group, the phenolic family includes over 8000 compounds [3,5]. Phenols, depending on their derivatives, are divided into three main groups: (a) phenolic acids that occur as hydroxylated benzoic acid derivatives; (b) phenolic acids as cinnamic acid derivatives; and (c) glycosidic phenylpropanoids [6]. There is an increasing interest in the biological effects of phenols, since there are to be found numerous studies connecting phenolic content and antioxidant, antimicrobial, and anticancer activities of fruits, vegetables, flowers, leaves, and seeds [7]. Antioxidants are chemical compounds that can quench reactive radical intermediates formed during oxidative reactions. The primary antioxidants comprise essentially sterically hindered phenols and secondary aromatic amines [8,9]. The majority of natural antioxidants are phenolic compounds [10].

Having around 900 species around the world, *Salvia* L. belongs to the Lamiaceae family and is used in flavoring, cosmetics, perfumery, the pharmaceutical industries, and folk medicine [11]. Up until now, different *Salvia* species have been investigated for their biological activity including anti-inflammatory [12], antioxidant-antifungal [13], anticancer [14], as well as antiviral activities [15]. A promising study also suggested that *Salvia lavandulifolia* Vahl. could be used for dementia therapies since its active components show anticholinesterase activity, which is a potent treatment for Alzheimer's disease [16]. In addition, *S. limbata*, *S. hypoleuca*, and *S. aethiopis* exhibited considerable cytotoxic activity against the tested three human cancer cell lines [17].

Pathogen infections have threatened human health for many years. During the past decades, with the abuse of antimicrobial agents, more and more drug-resistant pathogens have been found. Among them, *Staphylococcus aureus* is a prominent pathogen, which causes a public health concern worldwide and is associated with a high mortality [18,19]. Novel antimicrobial agents against methicillin-resistant *Staphylococcus aureus* have been introduced recently. However, the emergence of resistance and side effects for these agents increases the need for novel antimicrobial agents [20]. Bacterial species may cause food poisoning and their elimination from food is needed. *S. aureus* and *Escherichia coli* have been reported to lead to the poisoning found in ice cream and raw beef, respectively [21,22]. Fungal species, on the other hand, are related to infectious diseases. *Candida albicans* causes blood stream infection [23,24]. In addition *Saccharomyces cerevisiae* traditionally was considered as a harmless fungus, however recent studies suggested that it could be pathogenic in severely immune compromised patients, causing systemic infection [25]. In this study, antimicrobial and anti-fungal activities of the selected samples were evaluated against seven different microbial and three different fungi species, including the ones mentioned above.

The positive role of plants on human health as well as the pharmacological characteristics of the *Salvia* species is thought to be due to their phenolic acid contents. For example caffeic acid derivatives from *S. miltiorrhiza* have been suggested to be effective in the inhibition of liver fibrosis and hepatoprotection as well as be protective against cerebral and heart ischemia-reperfusion [26]. Furthermore, rosmarinic acid derivatives from *S. officinalis* showed potent antioxidant activity greater than a standard synthetic antioxidant, trolox [27]. Moreover, *S. fruticosa*, which is rich in luteolin and rutin, showed significant anti-inflammatory activity comparable to a standard drug [12]. Therefore, it is interesting to discover the constituents and biological activities of different *Salvia* species due to their pharmacological potential. 

Although there are extensive reports on the bioactive properties of *Salvia* species, there are still undiscovered endemic ones, which potentially could be sources of a specific phenol or indicate unique antioxidant and antimicrobial activities. In Turkey, there are 89 recorded *Salvia* species with 93 taxa, 45 of which are endemic. In this study, we used three *Salvia* species (*S. microstegia*, *S. aethiopis* and *S. brachyantha*) to evaluate their antioxidant and antimicrobial activities as well as their phenolic contents. For identification and quantification of phenolic compounds, a reverse phase high performance liquid chromatography (RP-HPLC) method coupled with mass spectrometry was used, which, in the literature, is a commonly used technique for separation of phenols [28]. Since phenolic content could be connected to antioxidant and antimicrobial activity, we also measured the antioxidant and antimicrobial activities of these *Salvia* species. Additionally, in this study we evaluated antioxidant activity based on reducing power capacity via ferric ions (Fe^3+^) reducing assay (FRAP) and cupric ions (Cu^2+^) reducing assay (CUPRAC); and radical scavenging activity via 1,1-diphenyl-2-picrylhydrazyl (DPPH) scavenging activity.

## 2. Materials and Methods

### 2.1. Plant Materials

The environmental conditions of the studied samples are as follows: *S. brachyantha* grows in open, calcareous stony igneous slopes, rocky ledges, and steppes at an altitude of 1400–1600 m. The vegetation in these places is mostly formed by herbaceous and woody plants including *Astragalus* sp., *Quercus* sp., *Verbascum* sp. and *Vicia* sp. *S. aethiopis* grows in open *Quercus* forests, steppes, igneous and limestone slopes, and fallow fields, along with herbaceous plants, such *S. multicaulis* Vahl and 

*S. trichoclada* Benth., species of *Trigonella*, *Astragalus*, *Alyssum*, *Galium*, *Vicia,* and *Bromus*, at elevations between 1200 and 1500 m. *S. microstegia* grows on calcareous rocky limestone and igneous slopes, cliffs, screes, fieldsides, and among *Quercus* shrubs at an altitude of 1300–1600 m. The vegetation in this place is formed by herbaceous and woody plants including *Quercus* spp., *Euphorbia* spp.

All plant samples were collected from their natural habitats by Dr. Omer Kilic. The first plant material, *S. brachyantha* was collected from the vicinity of Saban village (Bingöl) from rocky slopes, on 18 May 2014, at an altitude of 1400–1500 m, collection number; 5681. The second plant material, *S. aethiopis* was collected south of Yelesen village, from stony areas, on 20 June 2013, at an altitude of 1600–1700 m, collection number; 4780. The third plant material, *S. microstegia* was collected from east of Dikme village, from steppe and moist areas, on 5 September 2013, at an altitude of 1750–1800 m, collection number; 5405. All plant samples were identified by Kilic with Flora of Turkey and the East Aegean Islands [29]. The voucher specimens were deposited at the Department of Park and Garden Plants, Bingol University and Yıldırımlı Herbarium from Ankara. The samples were taken from the aerial parts of whole plants. The aerial parts of the studied samples were dried in a shady and airy place, held for one week.

### 2.2. Plant Extraction

Plants extractions were described previously in detail [30]. An ethanol extract was prepared by grinding 100 g air-dried samples in a mill and sample powders were mixed with 300 mL ethanol on a magnetic stirrer for 24 h at room temperature. Ethanolic extracts were filtered and the mixtures were then placed on a rotary evaporator at 30 °C to remove ethanol (Yield: 15.6%). For water extract preparation, sample powders were mixed with 100 mL of distilled water, filtered, and lyophilized under 5 μm Hg pressure at −50 °C. Samples were then stored in a tightly caped plastic bottle at −20 °C until used for experimental studies. 

### 2.3. HPLC Analysis

Dry filtrates were diluted to 1000 mg/L and filtered with a 0.2 µm microfiber filter prior to LC-MS/MS analysis [31]. LC-MS/MS analyses of the phenolic compounds were performed using a Nexera model Shimadzu UHPLC coupled to a tandem MS instrument (Shimadzu, Kyoto, Japan). The liquid chromatography was equipped with LC-30AD binary pumps (Shimadzu, Kyoto, Japan), a DGU-20A3R degasser (Shimadzu, Kyoto, Japan), a CTO-10ASvp column oven (Shimadzu, Kyoto, Japan), and a SIL-30AC auto sampler (Shimadzu, Kyoto, Japan). The chromatographic separation was performed on a C18 reversed-phase Inertsil ODS-4 (150 mm × 4.6 mm, 3 µm, GL Sciences, Tokyo, Japan) analytical column. The column temperature was fixed at 40 °C. The elution gradient consisted of mobile phase A (water, 5 mM ammonium formate and 0.1% formic acid) and mobile phase B (methanol, 5 mM ammonium formate, and 0.1% formic acid). The gradient program with the following proportions of solvent B was applied t (min), B%: (0, 40), (20, 90), (23.99, 90), (24, 40), (29, 40). The solvent flow rate was maintained at 0.5 mL/min and injection volume was set as 4 µL.

### 2.4. Mass Spectroscopy (MS) Instrumentation

MS detection was performed using a Shimadzu LC-MS 8040 model triple quadrupole mass spectrometer (Shimadzu, Kyoto, Japan) equipped with an electrospray ionization (ESI) source operating in both positive and negative ionization modes. LC-MS/MS data were collected and processed by Lab Solutions software (Shimadzu, Kyoto, Japan). The multiple reaction monitoring (MRM) mode was used to quantify the analyses: the assay of investigated compounds was performed following two or three transitions per compound, the first one for quantitative purposes and the second and/or the third one for confirmation. The optimum ESI conditions were determined as DL temperature; 250 °C, heat block temperature; 400 °C, nebulizing gas flow (nitrogen); 3 L/min and drying gas flow (nitrogen); 15 L/min [32].

### 2.5. Antioxidant Activity Studies 

#### 2.5.1. CUPRAC Assay

The reducing capacity of extracts was measured by the CUPRAC method [33]. In this assay, 0.25 mL CuCl_2_ solution (0.01 M), 0.25 mL of ethanolic neocuproine solution (7.5 × 10^−3^ M) and 0.25 mL of CH_3_COONH_4_ buffer solution (1.0 M) were mixed and sample extract at different concentrations (10–30 µg/mL) was added to this mixture. The final volumes were adjusted to 2 mL with distilled water and 30 min later absorbances of the samples were measured at 450 nm. Increased absorbance was interpreted as increased reducing capacity.

#### 2.5.2. FRAP Assay

This assay was used to measure the Fe^3+^ ion’s reducing power [34]. The samples at different concentrations in distilled water were mixed with phosphate buffer (2.5 mL, 0.2 M, pH 6.6) and potassium ferricyanide [K_3_Fe(CN)_6_] (2.5 mL, 1%) and incubated at 50 °C for 20 min. Then, 2.5 mL trichloroacetic acid (10%) and 0.5 mL of FeCl_3_ (0.1%) were added to the reaction mixture. The increases in the absorbance were spectrophotometrically measured at 700 nm as an indication of reducing capacity.

#### 2.5.3. DPPH Assay

Hydrogen or electron donating abilities of the samples were measured using DPPH assay [35]. Accordingly, purple colored DPPH solution (1 mM) prepared in ethanol was added to samples at different concentrations (10–30 µg/mL). The mixture was incubated at room temperature for 30 min. and the radical scavenging activity of the samples was measured spectrophotometrically at 517 nm against a blank. Decreased absorbance of the sample indicated the DPPH free radical scavenging capability.

### 2.6. Antimicrobial Activity

#### 2.6.1. Microorganisms

In this study, three Gram-positive bacteria (*Bacillus subtilis* ATCC 6633, *Staphylococcus aureus* ATCC 25923, and *Bacillus megaterium* DSM 32), four Gram-negative bacteria (*Enterobacter aerugenes* ATCC 13048, and *Escherichia coli* ATCC 11229, *Pseudomonas aeruginosa* ATCC 9027 and *Klebsiella pneumoniae* ATCC 13883), as well as three fungi species (*Candida albicans* ATCC 10231, *Yarrowia lipolytica*, and *Saccharomyces cerevisiae*) were used as test microorganisms. In addition, erythromycin (E-15), ampicillin/sulbactam (SAM-20), amikacin (AK-30), and rifampicin (RD-5) were also used as positive control.

#### 2.6.2. Microbiological Assay

The antimicrobial activities of extracts were detected by the disc diffusion method. 30, 60, and 90 μL of each extract was absorbed onto sterile discs, of 8 mm diameter. To inoculate the media for assay, a 1% rate of each microorganism from 10^6^ to 10^7^ CFU/mL suspensions was added to 15 mL sterile media (for bacteria Muller-Hintone agar, for yeast Sabouraud 2% glucose agar). Each of these inoculated mediums was poured into a Petri dish (9 cm) and left at +4 °C for 1 h. Subsequently discs prepared from samples were added to these inoculated medias and left again at +4 °C for 1 h. Four antibiotic standard discs were used as the positive controls. Sensitivity was deduced by comparing the inhibition zone diameter produced by the erythromycin (E-15), ampicillin/sulbactam (SAM-20), amikacin (AK-30) and rifampicin (RD-5). The Petri dishes were incubated at 35 °C for 18–24 h, except for *C. albicans* ATCC 10231, *Y. lipolytica*, and *S. cerevisiae* which were incubated at 27 °C. Inhibition zones were measured, calibrated, and recorded as the mean diameter of three replications in mm.

## 3. Results and Discussion 

### 3.1. Identification of Phenols

For centuries, plant extracts have been used to treat many diseases and their mode of action may well have been based on the phenolic compound content [36,37]. Phenolic compounds are the most widely occurring groups of phytochemicals and are of considerable physiological and morphological importance in plants as well as having strong antioxidant properties [38]. Numerous studies have connected the antioxidant, anti-inflammatory, anti-cancer, and antimicrobial activities of many plants, herbs, and species to their phenolic content. For example, the anti-inflammatory, antioxidant, and anticancer activities of *Suaeda fruticosa* were explained by its appreciable level of phenolic compounds (31.8 mg gallic acid equivalent (GAE)/g dried weight (DW)) [39]. Therefore identification and quantification of phenols from different sources is becoming increasingly important due to their potential application for treating diseases. 

The commonly used sample preparation technique for phenols is extraction with organic solvents, while spectrophotometric and chromatographic techniques are the main methods utilized in their identification and quantification [36,40]. In this study, we identified the phenolic acids of three *Salvia* species from Turkey, using the RP-HPLC/MS technique. According to our present knowledge, no studies have been reported regarding phenolic compounds from *S. microstegia*, *S. aethiopis* and *S. brachyantha* in the open literature. However, there are other studies reporting phenolic compounds of different *Salvia* taxa [11,17,41,42].

**Figure 1:**
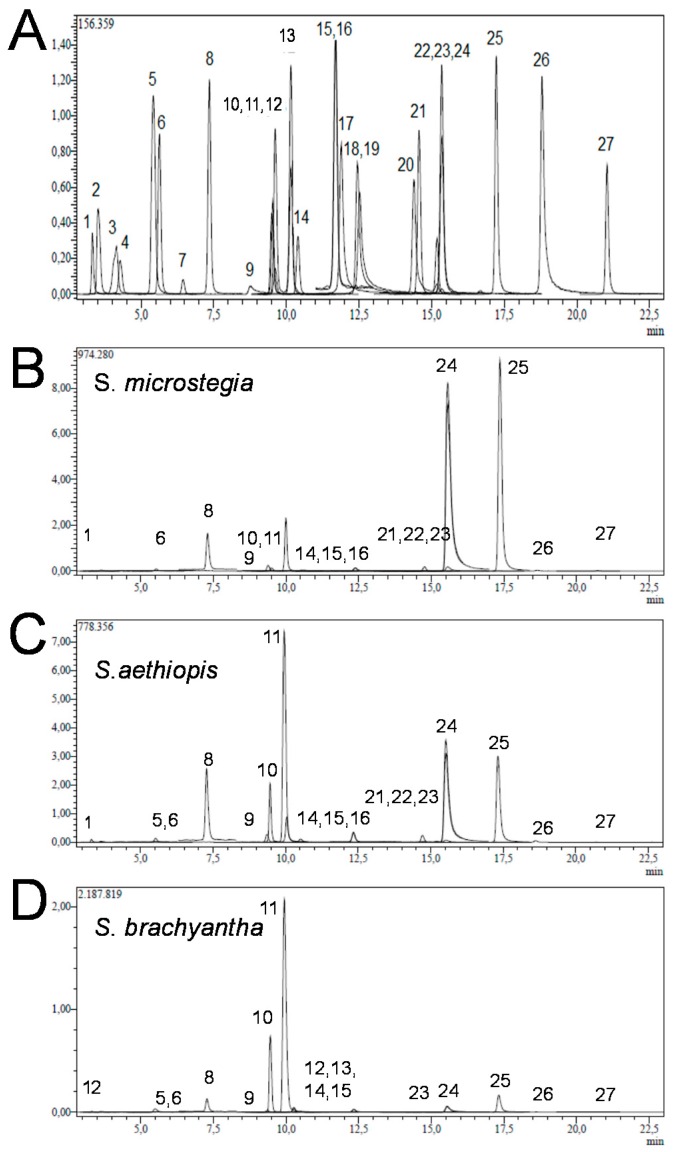
Typical HPLC chromatograms of (**A**) standards; (**B**) *S. microstegia*; (**C**) *S.aethiopis*; and (**D**) *S. brachyantha* where (1) is quinic acid, (5) chlorogenic acid, (8) trans-caffeic acid, (9) vanillin, (10) *p*-coumaric acid, (11) rosmarinic acid, (15) 4-hydroxybenzoic acid, (16) salicylic acid, (24) kaempferol and (25) apigenin. (For all compounds see Table 1).

The typical chromatograms of the samples are given in Figure 1. The identification of each peak was possible via retention time as well as MS spectra of samples and authentic standards. The characteristics of each peak from HPLC/MS analysis of authentic standards/samples are provided in Table 1. With 18 phenolic acids, *S. brachyantha* was found to be richest in terms of the number and amount of phenolic compounds.

In the analysis of HPLC/MS analysis, peak 1 exhibited a negative molecular ion at [MS−H]^+^ at *m*/*z* of 190.95 corresponding to quinic acid. Peak 2 had an *m*/*z* of 133.05 corresponding to malic acid. Peak 5 showed an *m*/*z* of 353, which indicates chlorogenic acid. Peak 6 had a negative molecular ion at an *m*/*z* of 152.95 and was identified as protocatechuic acid. Peak 8 indicated an *m*/*z* of 178.95, which corresponds to trans-caffeic. Peak 9 exhibited an *m*/*z* at 136.95 corresponding to vanillin. Peak 10 showed an *m*/*z* of 136.95 and the corresponding compound was identified as *p*-coumaric acid. Peak 11 exhibited an *m*/*z* of 151.05, which corresponds to rosmarinic acid. Peak 12 had an *m*/*z* of 353 corresponding to hesperidin. Peak 13 showed an *m*/*z* of 161.10, which indicates rutin. Peak 14 by HPLC-MS/MS gave a negative molecular ion at an *m*/*z* of 463.1 and was identified as hyperoside. Peak 15 indicated an *m*/*z* of 284.95, which corresponds to 4-hydroxybenzoic acid. Peak 16 exhibited an *m*/*z* at 136.95 corresponding to salicylic acid. Peak 21 showed an *m*/*z* of 270.95, which indicates naringenin. Peak 22 by HPLC-MS/MS gave a negative molecular ion at an *m*/*z* of 300.95 and was identified as hesperidin. Peak 23 indicated an *m*/*z* of 284.95, which corresponds to luteolin. Peak 24 exhibited an *m*/*z* at 284.95 corresponding to kaempferol. Peak 25 showed an *m*/*z* of 268.95 and the corresponding compound was identified as apigenin. Peak 26 exhibited a negative molecular ion at [MS − H]^+^ at *m*/*z* of 314.95 corresponding to rhamnetin. Peak 27 had an *m*/*z* of 253 corresponding to chrysin. Peak 5 showed an *m*/*z* of 358.90, which indicates chlorogenic acid.

### 3.2. Quantification of Phenols 

In order to establish the relationship between peak area and concentration, linear regression analysis was carried out for the investigated standards listed in Table 1. We constructed calibration curves in three different ranges (250–10,000 µg/mL, 25–1000 µg/mL, and 100–4000 µg/mL) for different standards. The linearity, which shows the correlation between the peak area and concentration, was expressed as correlation coefficient (*r^2^*) and higher than 0.999 for all standards except for hyperoside (*r*^2^: 0.950). We also calculated limit of detection (LODs) and limit of quantification (LOQs), which ranged from 0.05–25.8 to 0.17–74.5, respectively (Table 1) [43].

**Figure 2:**
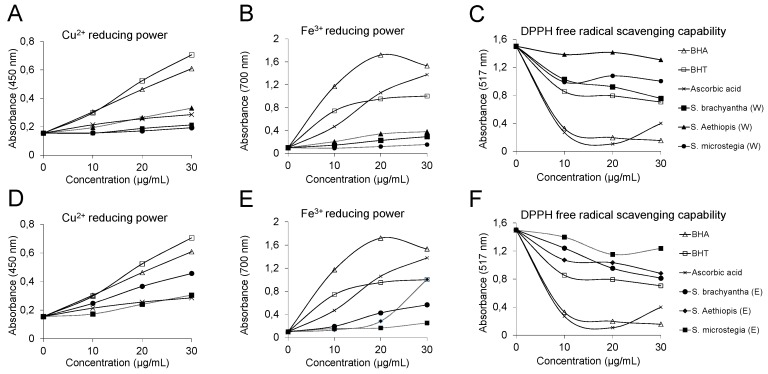
Antioxidant activities of three *Salvia* species and standards using (**A**,**D**) CUPRAC assay; (**B**,**E**) FRAP assay; and (**C**,**F**) DPPH assay (**E**: ethanolic extract, **W**: water extract).

With the aid of the established calibration curves of the standard compounds, we then quantified the phenolic compounds based on the area of individual peaks from the HPLC chromatogram profile of the samples and compared them with the areas of the standards at known concentrations. The amount of each compound was expressed as µg/kg dried *Salvia* extracts. The main phenolic acids were kaempferol in *S. microstegia* with 1954.28 µg/kg, rosmarinic acid in *S. brachyantha* with 7619.58 µg/kg, and *S. aethiopis* with 1904.53 µg/kg, respectively. By comparing Figure 2B,C and considering Table 2, it can be seen that, in terms of phenolic acid content, *S. microstegia* and *S. brachyantha* gave similar results. The less abundant phenolics were apigenin, *p*-coumaric acid, and luteolin for *S. microstegia*; kaempferol and apigenin for *S. aethiopis*; and chlorogenic acid and rutin for *S. brachyantha*. Differences in the variety and amount of phenolic compound from different *Salvia* species are expected since it is possible that the harvesting season and geographic origin affect the phenolic contents [44]. In addition, each species has a unique phenolic composition peculiar to that plant. For example, six different *Artemisia* species exhibited significant dissimilarities in terms of antioxidant capacities and phenolic compounds [45]. Furthermore, a study where eight *Salvia* species, namely *S. aethiopis*, *S. candidissima*, *Salvia chionantha* Vahl., *S. limbata*, *S. microstegia*, *S. nemorosa* L., *S. pachystachya* Trautv., *S. verticillata* L., and *S. virgata* Jacq., collected from Turkey were investigated for their amount of phenolic compounds, revealed that the amount of total phenolic content varies from 50.3 to 167.1 mg GAE/g DW among species [46]. Moreover, the extraction solution could be also affecting phenolic contents. For instance, the separate usage of water, 80% methanol, and 70% ethanol extracts of *Moringa oleifera* Lam. leaves resulted in 7.43%, 12.33%, and 11.04% in total phenolics, respectively [47].

### 3.3. Antioxidant Activity

An antioxidant molecule is a substance that, even at low concentration, can hinder or delay the oxidation of a substrate [48,49]. Hence there are multiple ways of measuring the antioxidant effect of a source to have complete understanding of the mechanism of action. Furthermore, an extract showing low antioxidant activity could not be labelled as a poor source of antioxidant, since an extract is composed of chemicals with different functional groups and polarities and may behave differently depending on the reaction mixture [50]. We used ethanol as well as water as extraction solvents to dismiss the possibility that some of the polyphenols are not extractable in water due to poor solubility [51]. In this study, the antioxidant capacity of three *Salvia* species were measured using FRAP, CUPRAC, and DPPH assays. 

The CUPRAC assay is widely used for measurement of the antioxidant capacity of plant extracts, due to the requirements of standard equipment, as well as fast and reproducible results [50]. This assay is based on the measurement of the absorbance of a complex that results from the reaction of antioxidant with Cu^2+^-neocuproine reagent [52]. The results showed that the cupric ions (Cu^2+^) reducing capacity of the ethanol extract of *S. brachyantha* was higher than the other samples tested, but lower than synthetic antioxidants butylated hydroxyanisole (BHA) and butylated hydroxytoluene (BHT) (Figure 2A). The water extract of *S. aethiopis* and the ethanol extract of *S. microstegia* showed an activity similar to ascorbic acid (Figure 2A,D). The lowest activity was observed in the water extract of *S. microstegia* (Figure 2A). It is worth noting that the ethanol extract of *S. microstegia* showed higher activity than the water extract, which might indicate that the ethanol extract has a higher phenolic content than the water extract. Similar findings were reported for *Salvia* species. For instance a previous study revealed that *Salvia chionantha* possessed lower CUPRAC values than BHA and α-tocopherol [53]. As mentioned above, another factor affecting the antioxidant capacity is the selection of extraction solvent, for example, ethyl acetate extract of *S. cadmica* indicated a CUPRAC value that was almost a third of the one where methanol was used as extraction solvent [11].

FRAP assay was used to measure the ferric ion (Fe^3+^) reducing capacity of the samples (Figure 2B). The overall results showed that all samples from both extraction solutions showed lower ferric ion reducing capacity than standard synthetic antioxidants (Figure 2B,E). The ethanol extract of *S. aethiopis* showed higher reducing power at a concentration of 30 µg/mL, the same as BHT. Similarly, *S. verticillata* has been previously shown to have a reducing activity less than synthetic antioxidant compounds (BHA and BHT) [41]. It was also shown that *Salvia* species could have different ferric ion reducing activities that could be classified as good, moderate, and poor antioxidant activity depending on the habitat in which they had been grown [54]. 

DPPH radical scavenging activity was also performed to gain a better understanding of the antioxidant activity of the samples [55]. An antioxidant molecule is able to reduce the stable radical DPPH, having a deep violet color in solution, to the yellow-colored 1,1-diphenyl-2-picrylhydrazyl (DPPH_2_) [56]. The results showed that ethanol and water extracts of all samples showed moderate radical scavenging activity when compared to standard antioxidants (Figure 2C,F). All of the *Salvia* species were able to donate electrons to neutralize the DPPH radical, showing a catalase-like, superoxide dismutase (SOD)-like activity [57]. The highest and lowest activities were observed in *S. brachyantha* and *S. aethiopis*, respectively. Among the samples tested *S. brachyantha* s showed a slightly better antioxidant activity in the CUPRAC and DPPH assay. The reason for this could be due to significant amounts of quinic and trans-caffeic acid found in *S. brachyantha* (Table 1). The findings of this study are consistent with the literature. It has been found that Brazilian *S. officinalis* has a DPPH scavenging activity comparable to gallic acid [57]. In addition *S. palaestina* essential oil showed an increasing DPPH radical scavenging activity in a concentration dependent manner (from 0.122 to 1.35 mg/mL), which was a higher weight concentration than the ones used in this study [58]. Different antioxidant capacities from different species are also to be expected. For instance, a study, where six *Salvia* species were tested for their antioxidant properties, showed that the DPPH radical scavenging activities of *S. aethiopis* and *S. candidissima* were 0% and 49.7%, respectively. 

### 3.4. Antimicrobial Activity 

Many microorganisms, which cause damage to human health, exhibit drug resistance due to inadequate use of antibiotics. Thus, there is a need for the discovery of new substances from natural sources, including plants. The in vitro antimicrobial activity by the agar disc diffusion method of extracts of *S. brachyantha*, *S. aethiopis*, and *S. microstegia* resulted in a range of growth inhibition patterns against pathogenic microorganisms given in Table 2. 

In this study, the strongest antibacterial activity was observed in the extracts of *S. brachyantha* and *S. microstegia* against *B. subtilis* (13 ± 0.47 mm inhibition zone) (Table 2). However the extracts of *S. brachyantha* and *S. microstegia* showed very weak antibacterial activity against *S. aureus* and *K. pneumoniae*. Meanwhile, the extract *S. aethiopis* extract did not show any antibacterial activity against *B. subtilis*, *B. megaterium*, and *K. pneumoniae*. The extract of *S. aethiopis* showed the highest antifungal activity against *Y. lipolytica* (13 ± 1.69 mm) and also showed no antifungal activity against *C. albicans* and *S. cerevisiae*, but the extract of *S. brachyantha* showed the highest antifungal activity against *C. albicans* and *Y. lipolytica* (10 ± 0.00 mm). The extracts did not show any antifungal activity against *S. cerevisiae* (Table 2).

It was observed that the antimicrobial effect of the plant extract varies from one plant to another in the different researches carried out in various regions of the world. This may be due to many factors such as, the effect of climate, soil composition, age, and vegetation cycle stage on the quality, quantity, and composition of extracted product of different bacterial strains [59,60].

The degree of antibacterial activity of the samples also changed with the increase in concentration of the samples. Roughly 1–2 mm increases were observed when the concentrations of samples were increased from 30, 60 to 90 µL. Antibacterial activities of *S. brachyantha*, *S. aethiopis*, and *S. microstegia* were also compared with reference antibiotics. As shown in Table 1, the extracts showed similar rates of activity with ampicillin/sulbactam (SAM-20 mg) and amikacin (AK-30 mg), but erythromycin (E-15 mg) and rifampicin (RD-5 mg) showed lower activity.

Similar findings could be found in the literature. For example *S. spinosa* exhibited potential antimicrobial effects against different tested Gram-negative and -positive strains [61]. Similarly, *Salvia chudaei* species was found to be effective against seven out of nine bacteria tested [42]. A different study showed that among eleven *Salvia* species, *S. eremophila*, *S. limbata*, *S. santolinifolia*, and *S. sclarea* indicated the highest antimicrobial activity whereas *S. aegyptiaca* and *S. aethiopis* showed the weakest activity against the tested organisms [17].

## 4. Conclusions

We investigated the antioxidant and antimicrobial activity of the phenolic compounds of three *Salvia* species from Turkey. This study is the first in which the identification and quantification of phenolic acids from *S. brachyantha*, *S. aethiopis* and *S. microstegia* have been accomplished. The results indicated that the methanol extract of S. *microstegia* contained considerable amounts of kaempferol while *S. brachyantha* and *S. aethiopis* were rich in rosmarinic acid. The findings of this study showed that all three species have reasonable and variable antioxidant activity depending on the method and extraction solution used. The compound extracted from *S. brachyantha*, *S. aethiopis*, and *S. microstegia* exhibited a broad spectrum of antimicrobial activity, which could be used as an alternative source for antibiotics. However, pharmacological testing is necessary to isolate and characterize the active compounds. These plant extracts should be investigated in vivo to better understand their safety, efficacy, and properties. Overall these species appear to be promising sources of various bioactive compounds that could be specifically used to treat certain diseases.

## Figures and Tables

**Table 1:** LC-MS/MS parameters of selected compounds and amount of three *Salvia* species (µg/kg).

No	Analyses	RT ^a^	Parent Ion (*m*/*z*) ^b^	Ionization Mode	*r*^2^ ^c^	RSD (%) ^d^	Linearity Range (µg/L)	LOD / LOQ (µg/L) ^e^	Recovery (%)	U ^f^	*S. brachyantha*	*S. aethiopis*	*S. microstegia*
1	Quinic acid	3.32	190.95	Neg	0.9927	0.0388	250–10,000	22.3 / 74.5	103.3	4.8	161.26	224.58	48.8
2	Malic acid	3.54	133.05	Neg	0.9975	0.1214	250–10,000	19.2 / 64.1	101.4	5.3	71.67	0	0
3	trans-Aconitic acid	4.13	172.85	Neg	0.9933	0.3908	250–10,000	15.6 / 51.9	102.8	4.9	ND	ND	ND
4	Gallic acid	4.29	169.05	Neg	0.9901	0.4734	25–1000	4.8 / 15.9	102.3	5.1	ND	ND	ND
5	Chlorogenic acid	5.43	353	Neg	0.9932	0.1882	250–10,000	7.3 / 24.3	99.7	4.9	355.05	25.42	0
6	Protocatechuic acid	5.63	152.95	Neg	0.9991	0.5958	100–4000	25.8 / 85.9	100.2	5.1	31.39	88.24	57.04
7	Tannic acid	6.46	182.95	Neg	0.9955	0.9075	100–4000	10.2 / 34.2	97.8	5.1	ND	ND	ND
8	trans-Caffeic acid	7.37	178.95	Neg	0.9942	1.0080	25–1000	4.4 / 14.7	98.6	5.2	151.77	289.68	176.8
9	Vanillin	8.77	151.05	Neg	0.9995	0.4094	250–10,000	10.1 / 33.7	99.2	4.9	40.75	52.28	28.91
10	*p*-Coumaric acid	9.53	162.95	Neg	0.9909	1.1358	100–4000	15.2 / 50.8	98.4	5.1	260.97	486.72	437.15
11	Rosmarinic acid	9.57	358.9	Neg	0.9992	0.5220	250–10,000	10.4 / 34.8	101.7	4.9	7619.58	1904.53	173.62
12	Hesperidin	9.69	611.1	Poz	0.9973	0.1363	250–10,000	21.6 / 71.9	100.2	4.9	354.74	ND	ND
13	Rutin	10.18	609.1	Neg	0.9971	0.8146	250–10,000	17.0 / 56.6	102.2	5.0	399.43	ND	ND
14	Hyperoside	10.43	463.1	Neg	0.9549	0.2135	100–4000	12.4 / 41.4	98.5	4.9	30.85	71.43	23.38
15	4-Hydroxybenzoic acid	11.72	136.95	Neg	0.9925	1.4013	25–1000	3.0 / 10.0	106.2	5.2	23.37	29.17	11.45
16	Salicylic acid	11.72	136.95	Neg	0.9904	0.6619	25–1000	4 / 13.3	106.2	5.0	19.96	26.33	11.08
17	Myricetin	11.94	317	Neg	0.9991	2.8247	100–4000	9.9 / 32.9	106.0	5.9	ND	ND	ND
18	Fisetin	12.61	284.95	Neg	0.9988	2.4262	100–4000	10.7 / 35.6	96.9	5.5	ND	ND	ND
19	Coumarin	12.52	146.95	Poz	0.9924	0.4203	100–4000	9.1 / 30.4	104.4	4.9	ND	ND	ND
20	Quercetin	14.48	300.9	Neg	0.9995	4.3149	25–1000	2.0 / 6.8	98.9	7.1	ND	ND	ND
21	Naringenin	14.66	270.95	Neg	0.9956	2.0200	25–1000	2.6 / 8.8	97.0	5.5	ND	28.91	22.55
22	Hesperetin	15.29	300.95	Neg	0.9961	1.0164	25–1000	3.3/ 11.0	102.4	5.3	ND	11.42	4.05
23	Luteolin	15.43	284.95	Neg	0.9992	3.9487	25–1000	5.8 / 19.4	105.4	6.9	25.41	171.50	437.46
24	Kaempferol	15.43	284.95	Neg	0.9917	0.5885	25–1000	2.0 / 6.6	99.1	5.2	129.92	764.82	1954.28
25	Apigenin	17.31	268.95	Neg	0.9954	0.6782	25–1000	0.1 / 0.3	98.9	5.3	222.54	416.76	1207.27
26	Rhamnetin	18.94	314.95	Neg	0.9994	2.5678	25–1000	0.2 / 0.7	100.8	6.1	2.50	25.19	12.89
27	Chrysin	21.18	253	Neg	0.9965	1.5530	25–1000	0.05 / 0.17	102.2	5.3	0.94	0.42	2.51

^a^ RT: retention time; ^b^ Parent ion (*m/z*): Molecular ions of the standard compounds (mass to charge ratio); ^c^ *r*^2^: coefficient of determination; ^d^ RSD: relative standard deviation; ^e^ LOD/LOQ (µg/L): limit of detection/limit of quantification; ^f^ U (%): percent relative uncertainty at 95% confidence level (*k* = 2); ND: not determined.

**Table 2:** Antimicrobial, and antifungal of activities of 20 µg/mL concentration of *Salvia* species against different microbial and fungal species (mm zone).

Microorganisms		*Salvia brachyantha*			*Salvia aethiopis*			*Salvia microstegia*			Antibiotic Discs (mm)			
		30	60	90	30	60	90	30	60	90	Erythromycin	Ampicillin/Sulbactam	Amikacin	Rifampicin
Gram-positive	*B.subtilis*	10 ± 0.00	11 ± 0.47	13 ± 0.47	-	-	-	11 ± 0.00	12 ± 1.69	13 ± 0.47	20 ± 1.24	14 ± 0.47	11 ± 1.24	21 ± 1.24
	*S. aureus*	9 ± 0.47	10 ± 0.81	11 ± 0.47	9 ± 0.47	11 ± 0.81	12 ± 0.00	9 ± 0.47	10 ± 0.47	11 ± 0.81	21 ± 0.00	10 ± 0.81	9 ± 0.00	18 ± 1.69
	*B. megaterium*	9 ± 0.00	10 ± 0.00	11 ± 0.00	-	-	-	9 ± 0.47	11 ± 0.00	12 ± 1.69	25 ± 1.69	-	10 ± 0.81	16 ± 1.24
Gram-negative	*E. aerogenes*	9 ± 0.81	10 ± 0.47	12 ± 0.81	-	9 ± 0.00	10 ± 0.47	9 ± 0.00	10 ± 0.81	11 ± 0.00	27 ± 1.24	10 ± 0.47	9 ± 0.00	16 ± 0.47
	*E. coli*	9 ± 0.47	10 ± 0.00	12 ± 1.69	-	9 ± 0.00	10 ± 0.00	9 ± 0.00	11 ± 0.81	12 ± 0.81	19 ± 0.00	13 ± 1.24	13 ± 0.81	18 ± 1.24
	*P. aeruginosa*	10 ± 0.00	12 ± 0.81	10 ± 0.47	-	9 ± 0.47	9 ± 0.47	9 ± 0.81	10 ± 0.00	11 ± 0.47	19 ± 1.69	-	14 ± 0.00	8 ± 0.00
	*K. pneumoniae*	9 ± 0.00	10 ± 0.47	10 ± 0.00	-	-	-	9 ± 0.00	10 ± 0.47	11 ± 0.00	19 ± 0.47	16 ± 1.69	10 ± 0.47	19 ± 1.69
Fungus	*Y. lipolytica*	-	-	10 ± 0.00	-	-	13 ± 1.69	-	-	-	-	-	-	-
	*C. albicans*	-	9 ± 0.00	10 ± 0.00	-	-	-	-	-	-	-	-	-	-
	*S. cerevisiae*	-	-	-	-	-	-	-	-	-	-	-	-	-
